# Myxedema Coma: A Life-Threatening Condition in Patients Using Pembrolizumab

**DOI:** 10.1155/2020/8855943

**Published:** 2020-10-22

**Authors:** Sangeetha Gummalla, Madhura Manjunath, Brian Phillips

**Affiliations:** ^1^Berkshire Medical Center, 725 North Street, Pittsfield, MA 01201, USA; ^2^UMass Memorial Medical Center, 55 North Lane Ave, Worcester, MA 01655, USA

## Abstract

The advent of immune checkpoint inhibitors has significantly improved the prognosis of patients with advanced malignancies. As we begin to understand these medications, multiple immune-related adverse effects (irAEs) have been found with these drugs, including endocrinopathies. Understanding the treatment-related adverse events of these medications is critical for clinical practice. Thyroid-related adverse effects usually occur within the first three months of treatment and rarely after eight months. It can manifest as an early onset of thyrotoxicosis, which is largely asymptomatic, followed by a rapid transition to hypothyroidism, requiring long-term levothyroxine substitution. We present a case in which our patient was found unresponsive, hypothermic, and with respiratory failure almost after completing a year of treatment with pembrolizumab. He had an initial mild elevation in thyroid-stimulating hormone (TSH) of 6.52, although with normal free thyroxine (T4) of 1.06, in his first three months of starting treatment which then rapidly progressed to a true myxedema coma. The infrequency with which this occurs makes it a diagnostic challenge.

## 1. Introduction

Myxedema coma is defined as severe hypothyroidism leading to decreased mental status, hypothermia, and other symptoms related to slowing of function in multiple organs. It is a medical emergency with high mortality rate. It is a rare presentation, likely due to the widespread availability of TSH assays, which allows practitioners to monitor their patients' hormonal balance closely. The acuity with which this occurs, however, along with the varied clinical presentations makes it a diagnostic challenge. We report a case of myxedema coma in a patient who presented at a very advanced stage while being on therapy with pembrolizumab. The increased immune response induced by the immune checkpoint inhibitors like pembrolizumab leads to irAEs, some of which are usually irreversible. The mechanism of thyroid-related adverse effects is still unclear and appears independent of thyroid autoantibodies but may include T cells, natural killer cells, and/or monocyte-mediated pathways and are frequently associated with anti-PD1 antibodies [1–5].

## 2. Case Presentation

Our patient is a 70-year-old male with a history of stage IV metastatic left lower lobe adenocarcinoma of the lung who was on palliative pembrolizumab therapy, 200 mg every three weeks starting from October 2018. He received 200 mg intravenously over thirty minutes in normal saline at the rate of 200 ml/hr. He was followed up with his oncologist for his thyroid function monitoring and did not have any symptoms of hypothyroidism at the onset of his presentation. His time course of TSH along with free T4 is shown in Table 1.

He was admitted to the hospital after experiencing hematemesis on 6^th^ March 2020 for which an esophagogastroduodenoscopy showed multiple esophageal ulcers and prepyloric ulcers. His TSH was checked during that admission and found to be 84.60. His chronic lymphedema had worsened which was thought to be from hypoalbuminemia. He was discharged to a nursing home on omeprazole. Within 4 days, he was found unresponsive and without a pulse at the nursing home. Return of spontaneous circulation was achieved in 10 minutes, and the patient was intubated in the field for airway protection and transferred to our hospital's intensive care unit with multiorgan failure. He was found to have hypothermia, bradycardia, and severe anasarca. Despite all aggressive measures with IV fluids, broad-spectrum antibiotics, and dynamic support, he continued to have significant multiorgan failure, including acute respiratory failure, circulatory failure complicated by lower extremity deep venous thrombosis, and advanced end-stage liver disease. He was requiring three vasopressor medications to maintain hemodynamic stability and continued to have severe lactic acidosis with an overall grave prognosis. His TSH at that time was found to be 171 with a free T4 of 0.38. Endocrinology was consulted, and he was diagnosed with myxedema coma. He was immediately started on intravenous (IV) levothyroxine and oral liothyronine. He was also given IV hydrocortisone to cover for adrenal insufficiency although that was thought less likely in this patient. Given his poor outcome, multiple discussions were held with his healthcare proxy regarding goals of care, and he was made comfortable and terminally extubated. He was started on lorazepam for comfort and passed away peacefully.

## 3. Conclusion

Myxedema coma is a rare presentation in current times given easy accessibility of TSH testing. But the acuity of its presentation makes it a challenge in the diagnosis and management. We are discovering that, with patients who are on immune checkpoint inhibitors, frequent monitoring of thyroid functions is essential. More careful monitoring is required when patients are experiencing illnesses that alter their normal physiological processes (such as having an infection and gastrointestinal bleed). Physicians should also educate their patients about these possible adverse effects. Endocrinopathies can often be managed by the treating oncologist or primary care physician; however, in more severe cases, an endocrinological evaluation and appropriate therapy may be needed. If not promptly recognized, endocrine dysfunction can quickly become life-threatening in such patients.

## Figures and Tables

**Table 1:** TSH and T4 values from Oct 2018 to March 2020.

Time	TSH	Free T4	Management
October 2018	2.21		Before starting pembrolizumab
November 2018	4.06	1.31	Not on treatment
December 2018–November 2019	1.74–3.12		Not on treatment
December 2019	6.52	1.28	Not on treatment
22^nd^ January 2020	13.8	1.25	On levothyroxine
12^th^ February 2020	26.50	0.99	On levothyroxine
03^rd^ March 2020	84.60		On levothyroxine and liothyronine

## Data Availability

The data used to support the findings of this study are available from the corresponding author upon request.

## References

[1] Almutairi A. R., McBride A., Slack M., Erstad B. L., Abraham I. (2020). Potential immune-related adverse events associated with monotherapy and combination therapy of ipilimumab, nivolumab, and pembrolizumab for advanced melanoma: a systematic review and meta-analysis. *Frontiers in Oncology*.

[2] Wang Y., Zhou Y. F., Qi X. (2019). Treatment-related adverse events of PD-1 and PD-L1 inhibitors in clinical trials: a systematic review and meta-analysis. *JAMA Oncology*.

[3] Mazarico I., Capel I., Giménez-Palop O., Albert L., Berges I. (2019). Low frequency of positive antithyroid antibodies is observed in patients with thyroid dysfunction related to immune check point inhibitors. *Journal of Endocrinological Investigation*.

[4] Ferrari S. M., Fallahi P., Galetta F., Citi E., Benvenga S., Antonelli A. (2018). Thyroid disorders induced by checkpoint inhibitors. *Reviews in Endocrine and Metabolic Disorders*.

[5] Su Q., Zhang X. C., Wang D. Y., Zhang H. R. (2018). The risk of immune-related endocrine disorders associated with anti-PD-1 inhibitors therapy for solid tumors: a systematic review and meta-analysis. *International Immunopharmacology*.

